# Corticosteroids do not influence the efficacy and kinetics of CAR-T cells for B-cell acute lymphoblastic leukemia

**DOI:** 10.1038/s41408-020-0280-y

**Published:** 2020-02-06

**Authors:** Shuangyou Liu, Biping Deng, Zhichao Yin, Jing Pan, Yuehui Lin, Zhuojun Ling, Tong Wu, Dong Chen, Alex H. Chang, Zhiyong Gao, Yanzhi Song, Yongqiang Zhao, Chunrong Tong

**Affiliations:** 1 Department of Hematology, Beijing Boren Hospital, Beijing, China; 2 Cytology Laboratory, Beijing Boren Hospital, Beijing, China; 3 Department of Bone Marrow Transplantation, Beijing Boren Hospital, Beijing, China; 4 Division of Hematopathology, Department of Laboratory Medicine and Pathology, Mayo Clinic, Rochester, MN USA; 5 Clinical Translational Research Center, Shanghai Pulmonary Hospital, Tongji University School of Medicine, Shanghai, China

**Keywords:** Cancer therapy, Haematological cancer

Dear Editor,

Chimeric antigen receptor (CAR) T-cell therapy has been demonstrated as a promising immunotherapeutic approach for treating the patients with relapsed/refractory B-cell acute lymphoblastic leukemia (B-ALL)^1–3^. However, cytokine release syndrome (CRS), the most prominent toxicity of CAR-T cell therapy, could be serious and even life-threatening^4,5^. Tocilizumab, an IL-6 receptor antagonist, has been widely used to treat CRS-related toxicities^5–7^, although corticosteroids could effectively abrogate CRS as well^4,5^, several earlier cases showed steroids may inhibit CAR T-cell persistence and their antimalignancy efficacy^7,8^, making corticosteroid therapy in CRS be often reserved for failure of tocilizumab or for neurologic toxicity since tocilizumab could not cross the blood-brain barrier. In our center, we use corticosteroids instead of tocilizumab as the first-line agent to manage CRS, here, we assessed the influence of steroids on the treatment effect and kinetics of CAR-T cells by comparing the difference between two groups of B-ALL patients who did (42 cases) or did not (26 cases) accept steroids.

Relapsed/refractory B-ALL^9^ patients treated by CAR-T therapy were from our clinical trials of ChiCTR-OIC-17013623 (CD19 for B-ALL), ChiCTR-ONC-17013648 (sequential CART for B-ALL after transplantation) and ChiCTR-OIC-17013523 (CD22 for B-ALL), these trials were approved by Beijing Boren Hospital institutional review board and informed consents were obtained. The lentiviral vectors encoding second generation CARs composed of CD3ζ and 4-1BB were used to produce CAR-T cells, a single dose or 2 fractionated doses (2 cases, within 3 days) of T-cells were infused. The construction of CD19- and CD22-specific CARs, the CAR-T cell manufacture and details of clinical protocols have been described in our published papers^10,11^ and on the ChiCTR website. Treatment effects were evaluated on day 30 after T-cell infusion and then monthly in follow-up patients. Minimal residual disease (MRD) was detected by multiparameter flow cytometry (FCM) and real-time quantitative PCR for fusion genes. Extramedullary diseases (EMD) were examined using PET-CT, CT or MRI. The dynamic monitoring of CAR-T cells was performed through flow cytometric quantitation of FITC + CD3 + T cells^12^. B-cell aplasia (BCA) was assayed by FCM and defined as less than 3% CD19- or CD22-positive lymphocytes^6^. *SPSS* 23 software was used to analyze data and tests were two-sided, comparison of means was performed using *T* test or Mann Whitney *U-*test when continuous variables were abnormal distribution, categorical variables were compared by the *chi*-square test. *P-*value < 0.05 was considered to be statistically significant.

Dexamethasone or methylprednisolone or both (alternately) were administrated for CRS when (1) continuous high fever not being released by antipyretics; (2) moderate-severe organ disfunction. Dexamethasone was applied in most cases especially for patients with neurologic symptoms; methylprednisolone was preferred for patients with pulmonary or liver dysfunction, and patients accepting high dose steroids. Steroids were usually started with low dose (dexamethasone 2–5 mg/dose) and would be increased if symptoms were not resolved, for severe CRS, steroids could be escalated up to dexamethasone 20 mg/m^2^/d or more higher up to methylprednisolone 10 mg/kg/d. Once CRS was improved, steroids were rapidly reduced and stopped. This study using corticosteroids to treat CRS was approved by Beijing Boren Hospital institutional review board.

A total of 68 patients were included, patients followed up less than 1 month and could not be evaluated (went to other hospitals for transplantation or died within 1 month) were excluded. The median age was 15 (range, 2–55) years, with 28 (41.2%) adults and 40 (58.8%) children younger than 18 years, 22 (32.4%) patients presented with EMD, bone marrow blasts in patients without EMD varied between 5%–96.5%. Thirty-one (45.6%) cases had an HCT. Fifty-four (79.4%) patients received CD19- and 14 (20.6%) received CD22-specific CAR-T therapy. CRS occurred in 94.1% (64/68) of patients, 10 (14.7%) cases experienced grade I, 44 (64.7%) experienced grade II and 10 (14.7%) experienced grade III CRS^13^. Five (7.4%) patients presented grade II (4 cases) and III (1 case) neurologic toxicity. Graft-versus-host disease (GVHD) induced by CAR-T therapy occurred in 6 (19.4%) of 31 post-hematopoietic cell transplantation (post-HCT) patients (Table 1A; Supplementary Table 1).

**Table 1:** Patient clinical data and treatment response.

Variables	No. (*n* = 68)	% of patients
**(A)** **Clinical data**		
Age		
Children (<18 years)	40	58.8
Adults	28	41.2
EMD		
Present	22	32.4
Absent	46	67.6
HCT		
Pre	37	54.4
Post	31	45.6
CAR-T type		
CD-19	54	79.4
CD-22^a^	14	20.6
CRS		
0	4	5.9
I	10	14.7
II	44	64.7
III	10	14.7
Neurotoxicity (≥ grade 2)	5	7.4
GVHD after CART in post-HCT patients (*n* = 31)	6	19.4
Steroids^b^		
Not received	26	38.2
Received	42	61.8
High dose^c^	23	54.8
≤7days	33	78.6
8–16 days	9	21.4

Treatment response	Steroid group (*n* = 42)		Non-steroid group (*n* = 26)		
	No.	%	No.	%	*p*-value
**(B) Treatment response on D30 after T-cell infusion**					
CR/CRi	40	95.2	24	92.3	0.344
MRD-CR	32	80.0	19	79.2	0.249
PR	2	4.8	1	3.8	
NR	0	0.0	1	3.8	

*EMD* extramedullary disease, *HCT* hematopoietic cell transplantation, *CRS* cytokine release syndrome, *GVHD* graft-versus-hos disease, *CR* complete remission, *CRi* CR with incomplete count recovery, *MRD* minimal residual disease, *PR* partial remission, *NR* no remission.

^a^14 patients, who failed or relapsed after CD-19 CART, or had dim CD-19 but normal CD-22 antigen expression, received CD-22 specific CAR-T therapy.

^b^Used within 1 month after T cell infusion, for all of 10 patients with grade III CRS, 68.2% (30/44) with grade II CRS, and 2 cases with no CRS but GVHD (1 case) or neurotoxicity (1 case).

^c^≥10 mg/m^2^/d dexamethasone or equivalent.

Within 1 month post CAR-T cell infusion, 42 (61.8%) patients were administrated steroids, including all of 10 with grade III CRS, 68.2% (30/44) with grade II CRS and 2 patients with no CRS but GVHD (1 case) or neurotoxicity (1 case), the duration of steroid use was 1-16 days (78.6% ≤ 7 days). Whereas 26 (38.2%) patients did not accept any steroids only supportive cares. Fourteen cases received CD22 CAR-T cells happened to be equally distributed in steroid and non-steroid group (each has 7 patients). In steroid group, 23 (54.8%) patients were given high-dose steroids (defined as ≥ 10 mg/m^2^/d dexamethasone or equivalent), the average days of high-dose steroid administration was 4 (range, 1-10 days) and 91.3% (21/23) of patients took high-dose steroids no more than one week (Table 1A; Supplementary Tables 1, 2). After one month, 3 patients continued to receive steroids against GVHD (2 cases, both lasting 4 months) or neurologic toxicity (1 case, lasting 17 days); 1 patient without using steroids within one month accepted 12-day steroids for GVHD, who was counted in the steroid group in later follow-up of B-cell aplasia.

We evaluated the impacts of steroids on treatment response. In the non-steroid group of 26 patients, 1 case had no response and 1 obtained partial remission (PR), the rest of 24 patients achieved complete remission (CR), the CR rate, composed of both CR and CR with incomplete count recovery (CRi)^9^, was 92.3% (24/26) and MRD negative CR was 79.2% (19/24). In the steroid group of 42 patients, 40 obtained CR and 2 were in PR, the rate of CR was 95.2% (40/42) and MRD negative CR was 80.0% (32/40). Obviously, there was no difference between steroid and non-steroid group in CR rate (*p* = 0.344) or in MRD^–^CR rate (*p* = 0.249) (Table 1B; Supplementary Table 1), this indicated that corticosteroids did not compromise the treatment effect of CAR-T cells for these patients.

Then, we analyzed the expansion of CAR-T cells in peripheral blood (PB) by assessing the quantity of CAR-T cells using FCM (Fig. 1a) on day 7, 11, 15, 20 and 30 after cell infusion (Fig. 1b; Supplementary Table 3). Unexpectedly, the average CAR-T cell numbers in steroid group were significantly higher than those in non-steroid group at all time points from day11 (*p* = 0.0302 on D11; *p* = 0.0053 on D15; *p* = 0.0045 on D20 and *p* = 0.0028 on D30), except for day 7 when CAR-T cells began to expand (*p* = 0.9815). These data demonstrated that steroids did not suppress the proliferation of CAR-T cells in PB, on the contrary, the T-cell expansion in steroid group was much greater, the reasonable explanation is that higher expansion of CAR-T cells led to the higher grade of CRS which hence needed to be controlled by steroids.

**Fig. 1:**
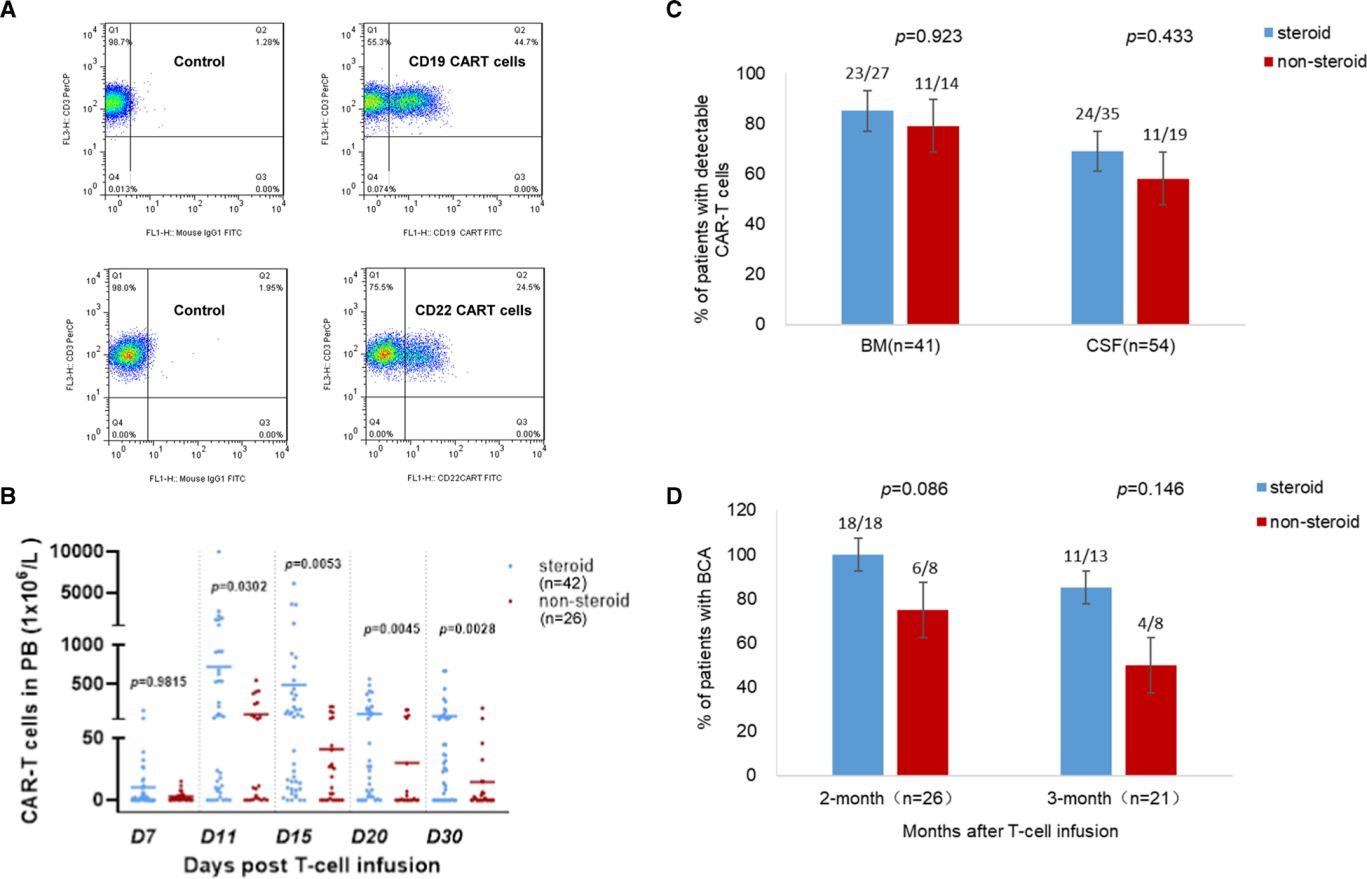
Kinetics of CAR-T cells in relapsed/refractory B-ALL patients treated or not treated with steroids (detected by flow cytometry). **a** The representative flow cytometry plots showing CAR-T cells. **b** CAR-T cell numbers in peripheral blood (PB) on day 7, 11, 15, 20 and D30. **c** Percentages of patients with detectable CAR-T cells in bone marrow (BM) and cerebrospinal fluid (CSF), assayed once or twice between day 14 to day 35. **d** Percentages of patients with B-cell aplasia (BCA) at 2 and 3 months. Based on Maude SL et al (N Engl J Med. 2014;371:1507-1517), BCA was defined as less than 3% CD19 or CD22 (4 cases) positive lymphocytes.

Since CAR-T cells can distribute to bone marrow (BM) and cerebrospinal fluid (CSF)^12,14,15^, we investigated the existence of CAR-T cells in BM and CSF by detecting them once or twice during day 14-35 after cell infusion. In steroid group, the percentages of patients with detectable CAR-T cells were 85.2% (23/27) in BM and 68.6% (24/35) in CSF; while in non-steroid group, the percentages of patients with CAR-T cells were 78.6% (11/14) in BM and 57.9% (11/19) in CSF, there were no significant differences between two groups (*p* = 0.923 in BM and *p* = 0.433 in CSF, respectively) (Fig. 1c; Supplementary Table 4). This implied that steroids did not interfere the trafficking of T-cells to BM and CSF.

The persistence of functional CAR-T cells was usually assessed by B-cell aplasia (BCA)^6,12^, we hence monitored BCA monthly in evaluable patients. Since most of this cohort of patients underwent transplantation or received other therapies after CART, BCA was only observed in some patients and the number of patients gradually decreased over time. At 2- and 3-month, 100% (18/18, 2 after CD22) and 84.6% (11/13, 1 after CD22) of patients presented BCA in steroid group, compared to 75% (6/8, 2 post CD22) and 50% (4/8, 1 post CD22) of patients in non-steroid group, the percentages of patients with BCA in steroid group were higher than those in non-steroid group but there were no significant differences between 2 groups (*p* = 0.086 at 2 months; *p* = 0.146 at 3 months) (Fig. 1d). In later time points of 4–6 months, although limited cases (all were after CD19) left in each group which could not be compared, in the steroid group, 100% of patients (4-month, 7/7; 5-month, 7/7; 6-month, 5/5) still maintained BCA and CR (Supplementary Table 5). This evidence indicated that the functional CAR-T cells could exist a longer time without being impacted by corticosteroids.

In conclusion, our data revealed that corticosteroids even high-dose steroids did not influence treatment outcomes of CAR-T cells and their proliferation and duration. The possibility of our results differing from previous observation could be: we used high-dose steroids for short term (average 4 days; 91.3% ≤ 7 days) whereas they used longer time (Davila ML et al mentioned 3 cases accepted 7-21 days of high-dose steroids^7^). Very recently, two studies showed that early intervention with corticosteroids for CRS^16^ or neurotoxicity^17^ does not impact on the antitumor potency of CD19 CAR -T cells, which support our results, the potential mechanisms need to be further explored.

## Supplementary information

supplimentary table1

supplimentary table2

supplimentary table3

supplimentary table4

supplimentary table5

ethics approval
