# Supplementary material for: Corticosteroids do not influence the efficacy and kinetics of CAR-T cells for B-cell acute lymphoblastic leukemia
Source: Blood Cancer J. 2020 Feb 6;10(2):15. doi: 10.1038/s41408-020-0280-y (PMC7005173; doi:10.1038/s41408-020-0280-y)
Supplement: Supplementary file 1 — supplimentary table1 [file 41408_2020_280_MOESM1_ESM.pdf]

**Table S1 Characteristics and treatment outcomes for individual patients**

| Pt.No. | Age | Pre-HCT | Blasts in BM(%) | EMD | CRS grade | Neurotoxicity(≥grade 2) | Days of steroid use within 1 month after T-cell infusion | | | Treatment response on D30 |
|---|---|---|---|---|---|---|---|---|---|---|
| | | | | | | | Total days | DXM≥10mg/m$^2$ or equivalent | DXM<10mg/m$^2$ or equivalent | |
| **1** | 15 | - | 43.2 | - | 2 | - | 7 | 5 | 2 | CR/MRD- |
| **2** | 2 | - | 10.0 | CNS | 2 | - | - | - | - | CR/MRD- |
| **3** | 4 | - | 95.5 | CNS | 2 | - | 3 | 3 | 0 | CR/MRD- |
| **4** | 24 | - | 67.5 | - | 2 | - | 7 | 0 | 7 | CR/MRD- |
| **5** | 6 | - | 46.5 | - | 3 | - | 5 | 5 | 0 | CR/MRD- |
| **6** | 5 | - | 25.0 | - | 3 | - | 12 | 7 | 5 | CR/MRD- |
| **7** | 17 | - | 90.0 | - | 3 | - | 16 | 4 | 12 | CR/MRD+(FCM 0.32%) |
| **8** | 7 | - | 90.0 | - | 2 | - | - | - | - | NR |
| **9** | 42 | - | 8.5 | - | 2 | - | 1 | 0 | 1 | CR/MRD- |
| **10** | 7 | - | 5.0 | - | 2 | - | - | - | - | CR/MRD- |
| **11** | 5 | - | 0.4 | Testis | 2 | - | 2 | 0 | 2 | CR/MRD- |
| **12** | 16 | - | 54.0 | - | 2 | - | 1 | 1 | 0 | CR/MRD- |
| **13** | 3 | - | 96.0 | - | 1 | - | - | - | - | CR/MRD- |
| **14** | 16 | - | 39.0 | - | 2 | - | 7 | 5 | 2 | CR/MRD- |
| **15** | 5 | - | 60.5 | - | 3 | 2 | 3 | 1 | 2 | CR/MRD- |
| **16** | 3 | - | 8.5 | - | 2 | - | 2 | 2 | 0 | CR/MRD- |
| **17** | 3 | - | 10.0 | - | 2 | - | - | - | - | CR/MRD- |
| **18** | 39 | - | 44.0 | - | 3 | - | 5 | 5 | 0 | CR/MRD- |
| **19** | 5 | - | 12.9 | - | 2 | 2 | 3 | 3 | 0 | CR/MRD- |
| **20** | 7 | - | 4.0 | CNS | 2 | - | - | - | - | CR/MRD- |
| **21** | 6 | - | 33.5 | - | 2 | - | 1 | 1 | 0 | CR/MRD- |
| **22** | 25 | - | 70.0 | - | 2 | - | 1 | 0 | 1 | CR/MRD- |
| **23** | 10 | - | 89.5 | - | 2 | - | - | - | - | CR/MRD- |
| **24** | 5 | - | 78.5 | - | 3 | - | 5 | 3 | 2 | CR/MRD- |
| **25** | 6 | - | 16.5 | - | 2 | 2 | 16 | 8 | 8 | CR/MRD+（E2A/PBX10.22%） |
| **26** | 15 | - | 12.0 | - | 2 | - | - | - | - | CR/MRD- |
| **27** | 5 | - | 0 | Testis | 1 | - | - | - | - | CR |
| **28** | 13 | - | 96.0 | - | 3 | - | 3 | 3 | 0 | CR/MRD- |
| **29** | 5 | - | 0 | CNS | 2 | - | - | - | - | CR/MRD- |
| **30** | 7 | - | 62.5 | - | 2 | - | 3 | 3 | 0 | CR/MRD- |
| **31** | 3 | - | 0 | CNS | 2 | - | - | - | - | CR/MRD- |
| **32** | 20 | - | 70.0 | - | 2 | - | 4 | 0 | 4 | CR/MRD- |
| **33** | 4 | - | 90.0 | - | 3 | - | 16 | 10 | 6 | CR/MRD- |
| **34** | 25 | - | 0.2 | Multiple | 2 | - | 2 | 0 | 2 | CR |
| **35** | 27 | - | 96.5 | - | 2 | - | 8 | 3 | 5 | CR/MRD- |

| | | | | | | | | | | |
|---|---|---|---|---|---|---|---|---|---|---|
| **36** | 31 | - | 11.0 | - | 2 | - | 12 | 5 | 7 | CR/MRD+（BCR/ABL0.04%） |
| **37** | 7 | - | 10.0 | CNS | 1 | - | - | - | - | CR/MRD- |
| **38** | 37 | + | 49.6 | CNS | 2 | - | - | - | - | CR/MRD- |
| **39** | 15 | + | 0 | Breast | 2 | - | 3 | 0 | 3 | CR |
| **40** | 55 | + | 37.0 | - | 2 | - | 3 | 0 | 3 | CR/MRD- |
| **41** | 45 | + | 36.5 | - | 1 | - | - | - | - | CR/MRD- |
| **42** | 10 | + | 26.0 | - | 2 | - | 3 | 1 | 2 | CR/MRD- |
| **43** | 2 | + | 90.5 | - | 2 | - | - | - | - | CR/MRD- |
| **44** | 20 | + | 23.5 | - | 2 | - | 6 | 0 | 6 | CR/MRD- |
| **45** | 27 | + | 0 | Breast | 1 | - | - | - | - | CR |
| **46** | 19 | + | 10.0 | - | 2 | - | - | - | - | CR/MRD- |
| **47** | 7 | + | 0 | Multiple | 2 | - | 2 | 0 | 2 | PR |
| **48** | 10 | + | 25.0 | - | 1 | - | - | - | - | CR/MRD- |
| **49** | 12 | + | 16.0 | - | 2 | 2 | 1 | 0 | 1 | CR/MRD- |
| **50** | 38 | + | 0 | Multiple | 1 | - | - | - | - | PR |
| **51** | 7 | + | 91.0 | - | 2 | - | 3 | 0 | 3 | CR/MRD- |
| **52** | 51 | + | 9.0 | - | 1 | - | - | - | - | CR/MRD- |
| **53** | 9 | + | 66.0 | - | 2 | - | 1 | 0 | 1 | CR/MRD- |
| **54** | 10 | + | 14.5 | - | 2 | - | 4 | 1 | 3 | CR/MRD- |
| **55** | 18 | + | 0 | Multiple | 2 | - | 2 | 0 | 2 | CR |
| **56** | 21 | + | 11.0 | - | 2 | - | - | - | - | CR/MRD+（BCR/ABL 0.33%） |
| **57** | 27 | + | 75.5 | Breast | 2 | - | - | - | - | CR/MRD- |
| **58** | 27 | + | 0 | Neck | 0 | - | 14 | 0 | 14 | CR |
| **59** | 15 | + | 36.5 | - | 2 | - | 12 | 0 | 12 | CR/MRD- |
| **60** | 29 | + | 0.4 | Multiple | 2 | - | 2 | 0 | 2 | PR |
| **61** | 18 | + | 19.0 | - | 0 | - | - | - | - | CR/MRD- |
| **62** | 28 | + | 2.7 | Multiple | 3 | - | 7 | 4 | 3 | CR |
| **63** | 29 | + | 56.0 | - | 3 | - | 10 | 2 | 8 | CR/MRD- |
| **64** | 43 | + | 0 | CNS | 0 | 3 | 8 | 0 | 8 | CR/MRD- |
| **65** | 20 | + | 0 | Multiple | 1 | - | - | - | - | CR |
| **66** | 55 | + | 32.0 | - | 0 | - | - | - | - | CR/MRD- |
| **67** | 16 | + | 0.8 | Multiple | 1 | - | - | - | - | CR |
| **68** | 31 | + | 15.5 | - | 2 | - | 4 | 0 | 4 | CR/MRD- |

Pt. patient, HCT hematopoietic cell transplantation, BM bone marrow, EMD extramedullary disease, CRS cytokine release syndrome, DXM dexamethasone, CNS central nervous system, CR complete remission, PR partial remission, NR non-remission, MRD minimal residual disease.
