# Supplementary material for: Corticosteroids do not influence the efficacy and kinetics of CAR-T cells for B-cell acute lymphoblastic leukemia
Source: Blood Cancer J. 2020 Feb 6;10(2):15. doi: 10.1038/s41408-020-0280-y (PMC7005173; doi:10.1038/s41408-020-0280-y)
Supplement: Supplementary file 2 — supplimentary table2 [file 41408_2020_280_MOESM2_ESM.pdf]

Table S2 Days of high-dose steroid use (*n*=23)

Pt.No.	Days of steroid use within 1 month after T-cell infusion		
	Total days	DXM⩾10mg/m^2 or equivalent	DXM＜10mg/m^2 or equivalent
**1**	7	5	2
**3**	3	3	0
**5**	5	5	0
**6**	12	7	5
**7**	16	4	12
**12**	1	1	0
**14**	7	5	2
**15**	3	1	2
**16**	2	2	0
**18**	5	5	0
**19**	3	3	0
**21**	1	1	0
**24**	5	3	2
**25**	16	8	8
**28**	3	3	0
**30**	3	3	0
**33**	16	10	6
**35**	8	3	5
**36**	12	5	7
**42**	3	1	2
**54**	4	1	3
**62**	7	4	3
**63**	10	2	8

Pt. patient, DXM dexamethasone.
