# Supplementary material for: Corticosteroids do not influence the efficacy and kinetics of CAR-T cells for B-cell acute lymphoblastic leukemia
Source: Blood Cancer J. 2020 Feb 6;10(2):15. doi: 10.1038/s41408-020-0280-y (PMC7005173; doi:10.1038/s41408-020-0280-y)
Supplement: Supplementary file 4 — supplimentary table4 [file 41408_2020_280_MOESM4_ESM.pdf]

Table S4 Detectable CAR-T cells in BM and CSF*

Steroid group (*n*=42)			Non-steroid group (*n*=26)		
**Pt.No.**	**BM**	**CSF**	**Pt.No.**	**BM**	**CSF**
**1**	-	+	**2**	N/A	N/A
**3**	N/A	N/A	**8**	N/A	N/A
**4**	+	-	**10**	N/A	N/A
**5**	N/A	-	**13**	-	+
**6**	N/A	N/A	**17**	N/A	-
**7**	+	-	**20**	N/A	-
**9**	+	+	**23**	N/A	-
**11**	+	+	**26**	N/A	N/A
**12**	N/A	+	**27**	+	+
**14**	N/A	+	**29**	N/A	-
**15**	+	+	**31**	N/A	+
**16**	N/A	N/A	**37**	+	-
**18**	+	-	**38**	-	N/A
**19**	N/A	+	**41**	-	-
**21**	N/A	-	**43**	N/A	N/A
**22**	+	+	**45**	+	-
**24**	+	+	**46**	N/A	N/A
**25**	N/A	+	**48**	N/A	+
**28**	N/A	+	**50**	+	+
**30**	N/A	+	**52**	+	+
**32**	+	+	**56**	+	+
**33**	N/A	+	**57**	+	+
**34**	+	+	**61**	+	-
**35**	-	-	**65**	+	+
**36**	+	+	**66**	+	+
**39**	N/A	N/A	**67**	+	+
**40**	+	+			
**42**	+	+			
**44**	+	-			
**47**	N/A	N/A			
**49**	N/A	-			
**51**	+	+			
**53**	-	N/A			
**54**	+	+			
**55**	+	+			
**58**	+	+			
**59**	+	-			
**60**	+	-			
**62**	+	+			
**63**	+	N/A			
**64**	-	-			
**68**	+	+			

BM bone marrow, CSF cerebrospinal fluid, Pt. patient.

*CAR-T cells were detected in BM and CSF by flow cytometry once or twice during D14 -D35 after T-cell infusion.
