# Supplementary material for: Corticosteroids do not influence the efficacy and kinetics of CAR-T cells for B-cell acute lymphoblastic leukemia
Source: Blood Cancer J. 2020 Feb 6;10(2):15. doi: 10.1038/s41408-020-0280-y (PMC7005173; doi:10.1038/s41408-020-0280-y)
Supplement: Supplementary file 5 — supplimentary table5 [file 41408_2020_280_MOESM5_ESM.pdf]

Table S5 B cell aplasia (BCA)* in later follow-up of 2-6 months

Month after T-cell infusion	Steroid group		Non-steroid group		*P*
	No. of patients	No.(%) with BCA	No. of patients	No.(%) with BCA	
**2**	18	18 (100)	8	6（75）	0.086
**3**	13	11 (84.6)	8	4（50）	0.146
**4**	7	7 (100)	1	1（100）	
**5**	7	7 (100)	1	1（100）	
**6**	5	5 (100)	0	0（ - ）	

*According to Maude SL et al (N Engl J Med. 2014;371(16): 1507-1517), B cell aplasia was defined as less than 3% CD19- or CD22-positive (4 cases) lymphocytes (assayed by flow cytometry).
