# Supplementary material for: Corticosteroids do not influence the efficacy and kinetics of CAR-T cells for B-cell acute lymphoblastic leukemia
Source: Blood Cancer J. 2020 Feb 6;10(2):15. doi: 10.1038/s41408-020-0280-y (PMC7005173; doi:10.1038/s41408-020-0280-y)
Supplement: Supplementary file 6 — ethics approval [file 41408_2020_280_MOESM6_ESM.pdf]

北京博仁医院医学伦理委员会批文
Beijing Boren Hospital Ethics Committee

北京博仁医院医学伦理委员会批文

BEIJING BOREN HOSPITAL ETHICS COMMITTEE/IRB APPROVAL FORM

批件编号（Number）	2017061504	会议日期(Meeting Date）	2017 年 6 月 15 日
项目名称<br>（Title）	糖皮质激素治疗 CART 相关性细胞因子释放综合征的临床研究<br>The clinical study of corticosteroids treating cytokine release syndrome (CRS) during CAR-T therapy		
临床中心<br>（Clinical Center）	北京博仁医院<br>Beijing Boren Hospital		
项目负责人<br>（Principal Investigator ）	童春容<br>Chunrong Tong		
审阅文件<br>（List of documents were reviewed）	1. 研究背景和可行性（research background and feasibility）<br>2. 研究方案（study protocol）		

投票结果：

同意（Approved）	修改后同意（Conditionally approved）	不同意（Disapproved）
9	0	0

会议结果<br>（Decision of meeting ）	■同意（Approved）<br>□修改后同意（Conditionally approved）<br>□不同意（Disapproved）

评价（Comments）：

经伦理委员会会议审查，同意研究者采用糖皮质激素治疗 CART 相关性细胞因子释放综合征。如果医疗中发生任何严重不良事件请立即通知本伦理委员会。

伦理委员会主任：吴彤

北京博仁医院医学伦理委员会（盖章）

北京博仁医院医学伦理委员会

日期：2017 年 06 月 15 日

地址：北京丰台区郑王坟南 6 号北京博仁医院 邮编：100070
