# Supplementary material for: Corticosteroids do not influence the efficacy and kinetics of CAR-T cells for B-cell acute lymphoblastic leukemia
Source: Blood Cancer J. 2020 Feb 6;10(2):15. doi: 10.1038/s41408-020-0280-y (PMC7005173; doi:10.1038/s41408-020-0280-y)
Supplement: Supplementary file 3 — supplimentary table3 [file 41408_2020_280_MOESM3_ESM.pdf]

Table S3 CAR-T cell numbers (×10^6/L) in PB*

Steroid group (*n*=42)						Non-steroid group (*n*=26)					
**Pt.No.**	**D7**	**D11**	**D15**	**D20**	**D30**	**Pt.No.**	**D7**	**D11**	**D15**	**D20**	**D30**
**1**	1.0	15.3	9.0	2.6	5.5	**2**	15.2	75.0	0.0	0.0	0.0
**3**	1.4	97.0	3540.0	566.0	45.0	**8**	3.5	404.0	135.0	170.0	46.0
**4**	1.1	920.0	84.0	100.0	45.0	**10**	7.6	1.7	0.0	0.0	32.6
**5**	0.4	7.6	0.0	0.0	0.0	**13**	11.4	99.0	0.0	0.0	0.0
**6**	15.6	910.0	175.0	2.6	0.0	**17**	0.3	54.0	10.0	3.7	0.0
**7**	38.9	10000.0	550.0	398.0	77.5	**20**	3.5	1.1	0.0	0.0	0.0
**9**	11.8	530.0	154.0	5.5	N/A	**23**	0.0	11.5	0.0	0.0	0.0
**11**	26.0	540.0	360.0	170.0	8.4	**26**	1.3	3.6	72.8	0.6	2.6
**12**	1.0	9.4	3.9	2.8	0.0	**27**	N/A	550.0	210.0	64.0	3.9
**14**	0.0	10.0	18.8	0.0	0.0	**29**	0.0	0.0	29.0	82.8	0.0
**15**	2.1	630.0	2.4	32.0	23.0	**31**	2.0	0.5	0.0	0.0	0.0
**16**	0.0	18.0	78.0	8.3	0.0	**37**	2.0	411.0	5.2	N/A	0.0
**18**	0.0	2770.0	170.0	46.0	0.0	**38**	0.0	0.0	1.3	0.0	0.0
**19**	0.0	1720.0	440.0	410.0	61.0	**41**	0.0	0.0	0.0	0.0	0.0
**21**	159.0	N/A	135.0	N/A	77.1	**43**	0.5	0.8	27.0	100.0	5.6
**22**	0.0	24.0	6120.0	N/A	278.0	**45**	0.0	0.0	40.6	0.0	0.0
**24**	4.6	1900.0	843.0	370.0	308.0	**46**	3.8	9.1	5.2	0.0	0.0
**25**	63.8	1710.0	117.0	N/A	86.2	**48**	4.5	N/A	0.0	170.0	59.0
**28**	0.3	66.5	110.0	N/A	440.0	**50**	0.0	10.0	150.0	N/A	190.0
**30**	0.0	11.6	5.4	7.7	0.0	**52**	3.4	260.0	210.0	N/A	0.0
**32**	32.5	101.0	7.9	N/A	51.0	**56**	0.1	75.6	59.9	29.7	15.1
**33**	0.0	1100.0	3600.0	57.0	57.0	**57**	7.8	243.0	18.9	6.8	1.6
**34**	0.0	22.0	15.0	27.4	670.0	**61**	0.7	N/A	27.0	1.0	1.7
**35**	0.0	0.0	0.0	0.0	0.0	**65**	0.0	0.0	0.0	0.0	0.0
**36**	0.0	680.0	29.3	24.0	36.3	**66**	0.9	378.0	44.0	N/A	15.2
**39**	0.0	2200.0	15.0	27.4	670.0	**67**	0.0	N/A	25.6	N/A	6.3
**40**	0.5	537.0	890.0	18.0	32.0						
**42**	10.0	920.0	13.7	128.0	83.0						
**44**	6.2	0.0	1.7	0.0	0.0						
**47**	27.0	210.0	386.0	193.0	110.0						
**49**	17.0	260.0	0.0	0.0	0.0						
**51**	0.0	0.4	40.0	143.0	37.0						
**53**	3.7	0.2	0.0	0.0	0.0						
**54**	0.0	0.0	16.3	487.0	26.0						
**55**	2.1	118.0	722.0	294.0	151.0						
**58**	0.0	N/A	305.0	N/A	13.8						
**59**	2.1	85.8	8.8	0.7	0.0						
**60**	0.0	5.9	120.0	N/A	11.5						
**62**	0.0	N/A	1240.0	213.0	181.0						
**63**	0.0	0.0	23.5	182.0	24.1						
**64**	2.4	2.4	10.0	13.0	0.4						
**68**	0.0	0.0	206.0	N/A	30.0						

PB peripheral blood, Pt. patient.

*CAR-T cells were assayed by flow cytometry, most of time, CAR-T cell numbers were detected exactly at each time point, a minority of patients were detected within ±1-2d and 8 cases were detected within ±3-5d of D30.
